# Hormonal therapy followed by chemotherapy or the reverse sequence as first-line treatment of hormone-responsive, human epidermal growth factor receptor-2 negative metastatic breast cancer patients: results of an observational study

**DOI:** 10.18632/oncotarget.14735

**Published:** 2017-01-18

**Authors:** Claudia Bighin, Beatrice Dozin, Francesca Poggio, Marcello Ceppi, Paolo Bruzzi, Alessia D’Alonzo, Alessia Levaggi, Sara Giraudi, Matteo Lambertini, Loredana Miglietta, Marina Vaglica, Vincenzo Fontana, Giuseppina Iacono, Paolo Pronzato, Lucia Del Mastro

**Affiliations:** ^1^ Department of Medical Oncology, IRCCS Azienda Ospedaliera Universitaria San Martino – Istituto Nazionale per la Ricerca sul Cancro, Genoa, Italy; ^2^ Department of Clinical Epidemiology, IRCCS Azienda Ospedaliera Universitaria San Martino – Istituto Nazionale per la Ricerca sul Cancro, Genoa, Italy

**Keywords:** advanced breast cancer, hormone-responsive breast cancer, first-line treatment, hormonal therapy, chemotherapy

## Abstract

Introduction Although hormonal-therapy is the preferred first-line treatment for hormone-responsive, HER2 negative metastatic breast cancer, no data from clinical trials support the choice between hormonal-therapy and chemotherapy.

Methods Patients were divided into two groups according to the treatment: chemotherapy or hormonal-therapy. Outcomes in terms of clinical benefit and median overall survival (OS) were retrospectively evaluated in the two groups. To calculate the time spent in chemotherapy with respect to OS in the two groups, the proportion of patients in chemotherapy relative to those present in either group was computed at every day from the start of therapy.

Results From 1999 to 2013, 119 patients received first-line hormonal-therapy (HT-first group) and 100 first-line chemotherapy (CT-first group). Patients in the CT-first group were younger and with poorer prognostic factors as compared to those in HT-first group. Clinical benefit (77 vs 81%) and median OS (50.7 vs 51.1 months) were similar in the two groups. Time spent in chemotherapy was significantly longer during the first 3 years in CT-first group (54-34%) as compared to the HT-first group (11-18%). This difference decreased after the third year and overall was 28% in the CT-first group and 18% in the HT-first group.

Conclusions The sequence first-line chemotherapy followed by hormonal-therapy, as compared with the opposite sequence, is associated with a longer time of OS spent in chemotherapy. However, despite the poorer prognostic factors, patients in the CT-first group had a superimposable OS than those in the HT-first group.

## INTRODUCTION

Breast cancer is not a single disease and it is now accepted that there are at least four different biological subtypes which comprise endocrine responsive disease divided in Luminal A (estrogen receptor [ER] and/or progesterone receptor [PgR] positive (+), HER2 negative (-), low proliferating activity) and Luminal B (ER and/or PgR+, HER2-, high proliferating activity), HER2+ disease (HER2+ regardless of hormone receptor status) and triple-negative disease (ER, PgR and HER2 negative [1–3].

The absolute majority of all newly diagnosed breast cancer patients (about 60-65%) [4] has hormone receptor positive (HR+) tumors; despite the best prognosis, still many patients relapse after treatment, forming the most common subtype of metastatic breast cancer (MBC) patients [5].

With current treatments, the median overall survival of HR+ MBC patients is estimated to be greater than 50 months [6] and it is crucial to plan the best sequence of treatments as it is expected that these patients receive multiple lines of therapy.

Most of HR+ MBC patients are treated with both hormonal therapy (HT) and chemotherapy (CT) during the course of their disease.

International guidelines indicate that HT is the preferred first-line treatment option for these patients [7–10] restricting the use of CT only to patients not responsive to HT or at risk of visceral crisis. However, this indication derives mostly from expert opinions and from very few randomized controlled trials.

A systematic review of trials comparing HT with CT as first-line treatment of MBC showed no significant difference in overall survival (OS) between the two types of treatment and an increased toxicity associated with CT [11]. However, no modern drugs, such as aromatase inhibitors or taxanes, were used in these trials and patients included in the CT arms did not receive maintenance HT after CT, which is now currently administered, although without a strong level of evidence [12].

Therefore, there are no strong data supporting that delivering first HT and CT thereafter provides a better outcome in terms of OS as compared to the reverse sequence.

The aim of the present study is to retrospectively compare the two treatment sequences in terms of objective response rate (ORR), clinical benefit rate (CBR), progression-free survival (PFS), OS, CT duration and time spent in CT (related to OS) in HR+/HER2- MBC patients.

## MATERIALS AND METHODS

All consecutive MBC patients treated with a first-line therapy (CT or HT with or without biological therapy) from 1999 to 2013 were included in this monocentric retrospective observational study. Patients were classified in four subgroups according to the biological characteristics of their primary tumor: Luminal A (ER and/or PgR+, HER2- and Ki67 < 20%), Luminal B (ER and/or PgR+, HER2- and Ki67 ≥ 20%), HER+ (immunohistochemistry score of 3+ or fluorescence in situ hybridization amplified regardless of hormonal status), and Triple-Negative (ER and PgR and HER2 negative) [13].

For the present study, we selected only Luminal A and B patients treated with at least one line of HT or one line of CT. Eligible patients were divided into two groups: the HT-first group including patients receiving HT as first-line treatment and the CT-first group including patients receiving CT as first-line treatment.

The choice between first-line HT or CT was performed by the treating physician on the basis of patients’ and disease characteristics.

Patients’ characteristics, histological and biological data from primary tumor, treatment history with clinical response at each line of treatment, PFS and survival data were collected.

### Statistical analysis

Patient and disease characteristics were analyzed using descriptive statistics. For each prognostic factor, heterogeneity between the two study groups was estimated by the Pearson Chi-square test or the Mann-Withney test.

Clinical response to therapy was assessed at the end of the first-line of treatment by clinical examination and imaging procedures, and classified according to RECIST guidance [14]. Objective response was defined as complete response (CR) if there was no evidence of residual tumor at the metastasis (MTS) site(s), partial response (PR) if the MTS mass was reduced by at least 50%. An increase in MTS size by 25% (as compared to baseline measurement) or new MTS appearance was considered as disease progression (PD). If the criteria for objective response or progression were not met, the disease was considered as stable (SD). Patients who experienced CR, PR or SD were considered as having obtained a CB from the therapy.

OS was estimated from the date of MTS diagnosis to the date of last contact or death from any cause.

Distant disease-free survival (DDFS) was estimated from the date of diagnosis of the primary tumor and the date of MTS diagnosis.

PFS was estimated from the date of first-line MBC therapy to the date of first subsequent PD.

Survival curves were obtained using the Kaplan-Meier method and differences between treatment sequences were assessed by the log-rank test [15].

Cox regression models were used for multivariate analysis to assess the independent role of each prognostic factor with respect to OS, while adjusting for the effects of the other factors. The variables included in the model as covariates were patient age at the time of relapse, menopausal status, tumor stage at diagnosis, sequence of systemic treatment (HT-first vs CT-first), median DDFS, presence of de novo metastatic disease, number of MTS sites, presence of visceral MTS (liver, lung and/or brain), presence of non-visceral MTS (bone, lymph node, skin and/or others). The multivariate analyses started from the full model, with all covariates included. Factors not significantly associated with survival were removed from the model by means of a step-down procedure based on the likelihood ratio test.

In subgroup analyses, effects of the CT-first sequence as compared to the HT-first sequence across the strata of each covariate were assessed by introducing the appropriate interaction terms in the Cox model. These covariates by treatment interaction terms were introduced in the model one at a time. The likelihood ratio test was used to evaluate the statistical significance of each interaction term.

Logistic regressions were used to predict a relationship between the use of CT as first-line treatment and the following factors: patient age at the time of MTS diagnosis, menopausal status, presence of de novo metastatic disease, number of MTS sites and type of MTS (visceral versus non-visceral). All statistical analyses were two-sided and were carried out using the SPSS package (version 18.0 for Windows). Significance was accepted for P values < 0.05.

With the aim of calculating the time spent in CT with respect to OS in the two patients groups, the proportion of patients in CT relative to those present in either group was computed at every day from the start of therapy. To adjust for OS, the proportions of patients in CT were weighted using as weights the survival function of the two groups. The weighted proportions were summed along time to estimate the cumulative average proportion of time patients spent in CT until then. For each proportion the 95% confidence intervals, based on the binomial distribution, were computed. LOWESS smoothing method has been applied to highlight the trend of proportions [16].

## RESULTS

**Figure 1:**
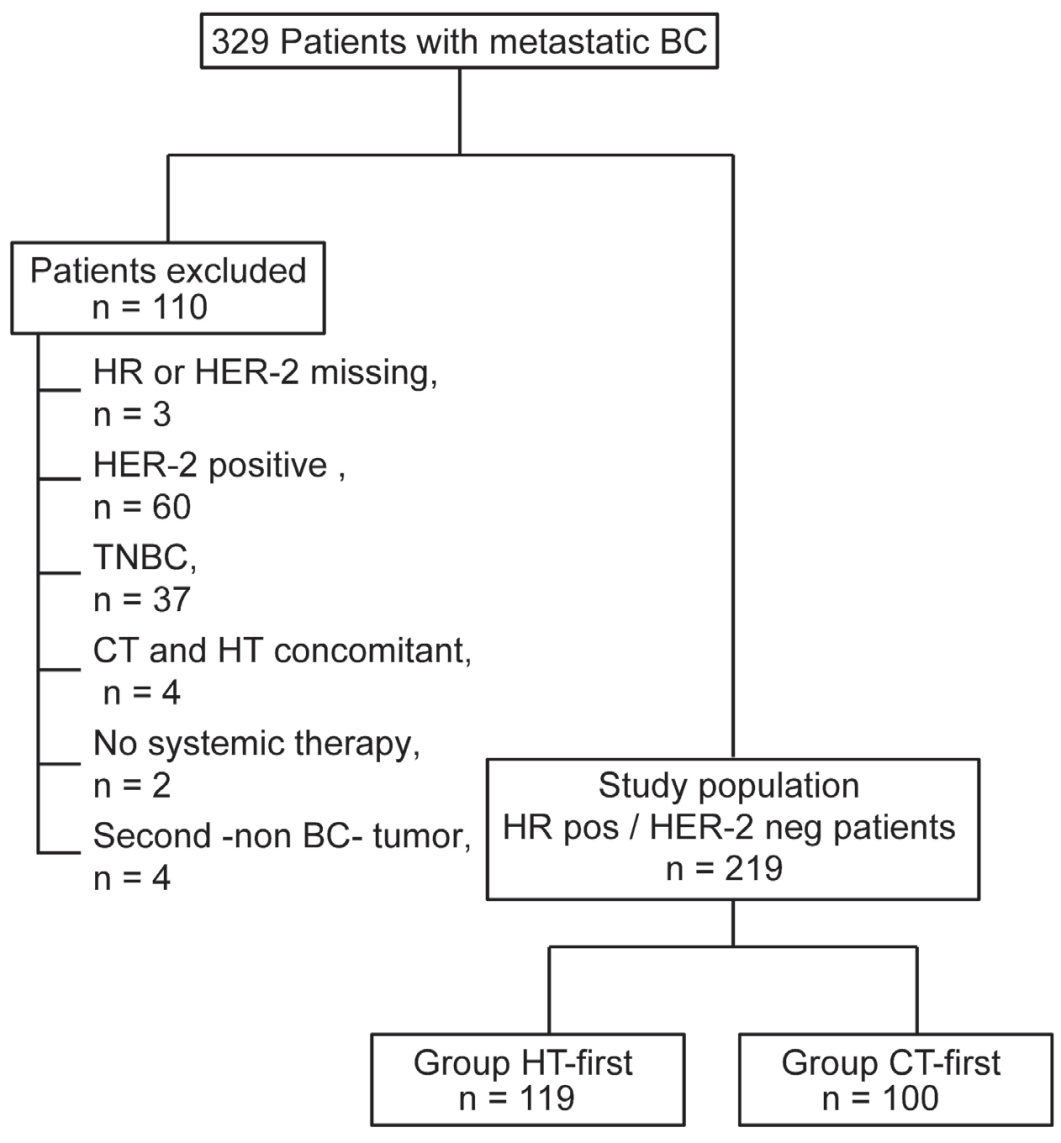
Flow-chart for the selection of the patient population to be included in the study

Three hundred twenty-nine patients with MBC were identified and 219 were included for this analysis

(Figure 1). Of the 110 patients who were excluded 60 were HER2+, 37 were triple-negative, 4 patients received CT concomitantly with HT as first-line treatment, 4 patients had a second non-breast tumor before the diagnosis of MTS and 2 patients did not receive any systemic therapy for their disease.

One hundred nineteen patients (54%) with hormone-responsive HER2- MBC received HT as first-line treatment with or without following CT (HT-first group) while 100 (46%) patients were treated with a first-line CT with or without following HT (CT-first group).

Table 1 summarizes patients and disease characteristics.

**Table 1:** Patient and disease characteristics by treatment group

	HT-first group (%)	CT-first group (%)	*p*^¶^
Total number of patients	119 (54)	100 (46)	
***Median age – year (range)*** At primary tumor diagnosis At metastasis diagnosis	61 (33-93) 67 (37-93)	54 (32-82) 57 (32-85)	0.049 <0.001
***Menopausal status*** Premenopausal Postmenopausal	31 (26) 88 (74)	45 (45) 55 (55)	0.003
***Stage at diagnosis*** I II III missing	19 (19) 35 (35) 44 (44) 2 (2)	14 (22) 17 (27) 32 (51) -	0.454
***De novo metastatic disease (stage IV)*** Yes No	19 (16) 100 (84)	37 (37) 63 (63)	0.001
***Adjuvant/Neoadjuvant CT***# Anthracycline alone / or + other Taxane alone / or + other Anthracycline and taxane alone / or+ other Other None	4 (4) 19 (19) 21 (21) 25 (25) 31 (31)	- 31 (49) 14 (22) 18 (29) -	<0.001
***Adjuvant/Neoadjuvant HT***# Only Tamoxifen Aromatase Inhibitors Tamoxifen →Aromatase Inhibitors None Missing	49 (49) 26 (26) 12 (12) 12 (12) -	28 (45) 14 (23) 10 (15) 10 (15) 1 (2)	0.570
***Disease-free survival*** #***, months*** ≤12 >12 – 36 >36	6 (6) 28 (29) 64 (65)	2 (3) 16 (25) 45 (72)	0.599
***Number of metastatic sites*** 1 2 ≥ 3	76 (64) 24 (20) 19 (16)	45 (45) 23 (23) 32 (32)	0.008
***Sites of metastasis*** Liver – yes no Lung – yes No Liver and Lung – yes No Brain – yes no Bone – yes no Lymph node – yes no	14 (12) 105 (88) 15 (13) 104 (87) 3 (2) 116 (98) 1 (1) 118 (99) 79 (67) 40 (33) 46 (39) 73 (61)	35 (35) 65 (65) 25 (25) 75 (75) 7 (7) 93 (93) 3 (3) 97 (97) 60 (60) 40 (40) 41 (41) 59 (59)	<0.001 <0.001 0.114 0.234 0.328 0.724

¶P values: from Pearson Chi-square test for categorical variables; from Mann-Withney test for continuous variable (age); #Metastatic ab initio excluded; Abbreviations: HT: hormonal-therapy; CT: chemotherapy

Median age at MTS diagnosis was 64 years (range 32-93); patients in the CT-first group were younger (median age 57 versus 67 years, *p* < 0.001) and more frequently in pre-menopausal status (45% versus 26%, *p* = 0.003) as compared to patients in the HT-first group.

Patients in the CT-first group were initially diagnosed at a more advanced stage as compared to patients in the HT-first group (37% versus 16% in stage IV, *p* = 0.0014) but DDFS between the primary tumor and the appearance of MTS was similar in the two treatment groups (*p* = 0.599). As expected, patients in the CT-first group had more metastatic sites (≥3 in 32% versus 16%, *p* = 0.008) and a greater liver or lung involvement than patients in the HT-first group (35% and 25% versus12% and 13%, respectively, *p* < 0.001). Nevertheless, the occurrence of concomitant liver and lung metastases was similarly low in the two treatment groups (2% and 7%, *p* = 0.114).

Considering only patients with stage I to III primary tumors, delivery of neo/adjuvant chemotherapy regimen differed between the two groups (*p* < 0.001): in particular, taxane alone or in combination with other drugs were delivered more often in the CT-first group than in the HT-first group (49% versus 19%). All patients in the CT-first group underwent at least one type of CT regimen while about one third (31%) of patients in the HT-first group did not receive any.

Neo/adjuvant hormonal therapy was given to a similar proportion of patients in the two treatment groups.

At the time of this analysis, in the HT-first group 42 patients (35%) did not have yet received any CT and, conversely, in the CT-first group 23 patients (23%) did not have yet receive any HT (Table 2).

**Table 2:** MBC therapy characteristics by treatment group

	HT-first group (%)	CT-first group (%)	*p*^¶^
***Median number of HT lines (range)***	2 (1-5)	1 (0-5)	<0.001
***Total number of HT lines*** 0 1 2 3 >3	0 (0) 119 (100) 80 (67) 48 (40) 33 (28)	23 (23) 77 (77) 45 (45) 24 (24) 11 (11)	
***Median total HT duration, months (range)***	20.7 (1.8-200.7)	19.3 (0.5-116.8)	0.692
***Median number of CT lines (range)***	1 (0-6)	2 (1-7)	<0.001
***Total number of CT lines*** 0 1 2 3 4 > 4	42 (35) 77 (68) 56 (47) 35 (29) 13 (11) 18 (15)	0 (0) 100 (100) 56 (56) 36 (36) 24 (24) 17 (17)	
***Median total CT duration, months (range)***	11.3 (0.2-38.2)	8.3 (0.03-53.2)	0.318
***First line chemotherapy*** Polychemotherapy Monochemotherapy None Missing	26 (22) 51 (43) 42 (35) 0 (0)	56 (56) 43 (43) 0 (0) 1 (1)	0.004
***Type of CT at first line*** Anthracycline alone Taxane alone Anthracycline and taxane Taxane and other Other No CT	2 (2) 12 (10) 9 (8) 12 (10) 42 (35) 42 (35)	9 (9) 16 (16) 27 (27) 19 (19) 29 (29) 0 (0)	0.004
***Type of HT at first line*** Tamoxifen Non-steroidal aromatase inhibitor Steroidal aromatase inhibitor Fulvestrant Everolimus Other No HT	11 (9) 57 (48) 19 (16) 27 (23) 1 (1) 4 (3) 0 (0)	13 (13) 43 (43) 9 (9) 11 (11) 1 (1) 0 (0) 23 (23)	<0.001

¶*P* values: from Pearson Chi-square test for categorical variables; from Mann-Withney test for continuous variable; Abbreviations: HT: hormonal-therapy; CT: chemotherapy

The median numbers of lines of treatments (HT plus CT) was similar in the two groups: 4 (range 1-10) in HT-first group and 3 (range 1-11) in CT-first group. No significant difference in the total duration of HT was observed between the two groups (median duration: 20.7 and 19.3 months in the HT-first and CT-first groups, respectively, *p* = 0.692). The total CT duration was similar in the two treatment groups (11.3 and 8.3 months in the HT-first and CT-first groups, respectively, *p* = 0.318).

A maintenance HT after first-line CT was administered in 63 patients (63%) in the CT-first group and letrozole was the most widely used drug (about 52% of patients).

Patients in the CT-first group received more frequently a poly-CT as first-line CT than patients in HT-first group (56% versus 22%). Among those patients in the CT-first group who received poly-CT, about one third (18 out of 56) had de novo metastatic disease. Poly-CT consisted of the combination of an anthracycline and a taxane more often in the CT-first group than in the HT-first group (27% versus 8%).

The most commonly drug used as first-line HT was a non-steroidal aromatase inhibitor (letrozole or anastrozole) in both groups (48% versus 43%) while fulvestrant was more frequently used as first-line HT in the HT-first group as compared to the CT-first group (23% versus 11%).

Only 2 patients (1 in HT-first group and 1 in CT-first group) received everolimus in association with exemestane.

### Objective response and clinical benefit

The ORR (CR or PR) observed after the first-line HT was not statistically different between the two sequence groups (19.8% and 30.6% in the HT-first group and in the CT-first group, respectively, *p* = 0.094). A similar result was observed when considering the first-line of CT (27.5% and 38.9% in the HT-first and the CT-first groups, respectively, *p* = 0.134).

No significant differences between the two treatment groups were observed in terms of CBR either after the first-line of HT (75.9% versus 79.2%, *p* = 0.6) nor the first-line of CT (73.9% versus 83.3%, *p* = 0.147).

### PFS and overall survival

Median follow-up was 3.14 years (range 0.12-16.70) and was similar for the two groups (3.31 years for the HT-first group and 2.89 years for the CT-first group, *p* = 0.246).

PFS after the first-line treatment was comparable in the two groups, being 13.4 months (range 2.3-74.4) for the HT-first group and 13.9 months (range 1.6-114.0) for the CT-first group (*p* = 0.911).

**Figure 2:**
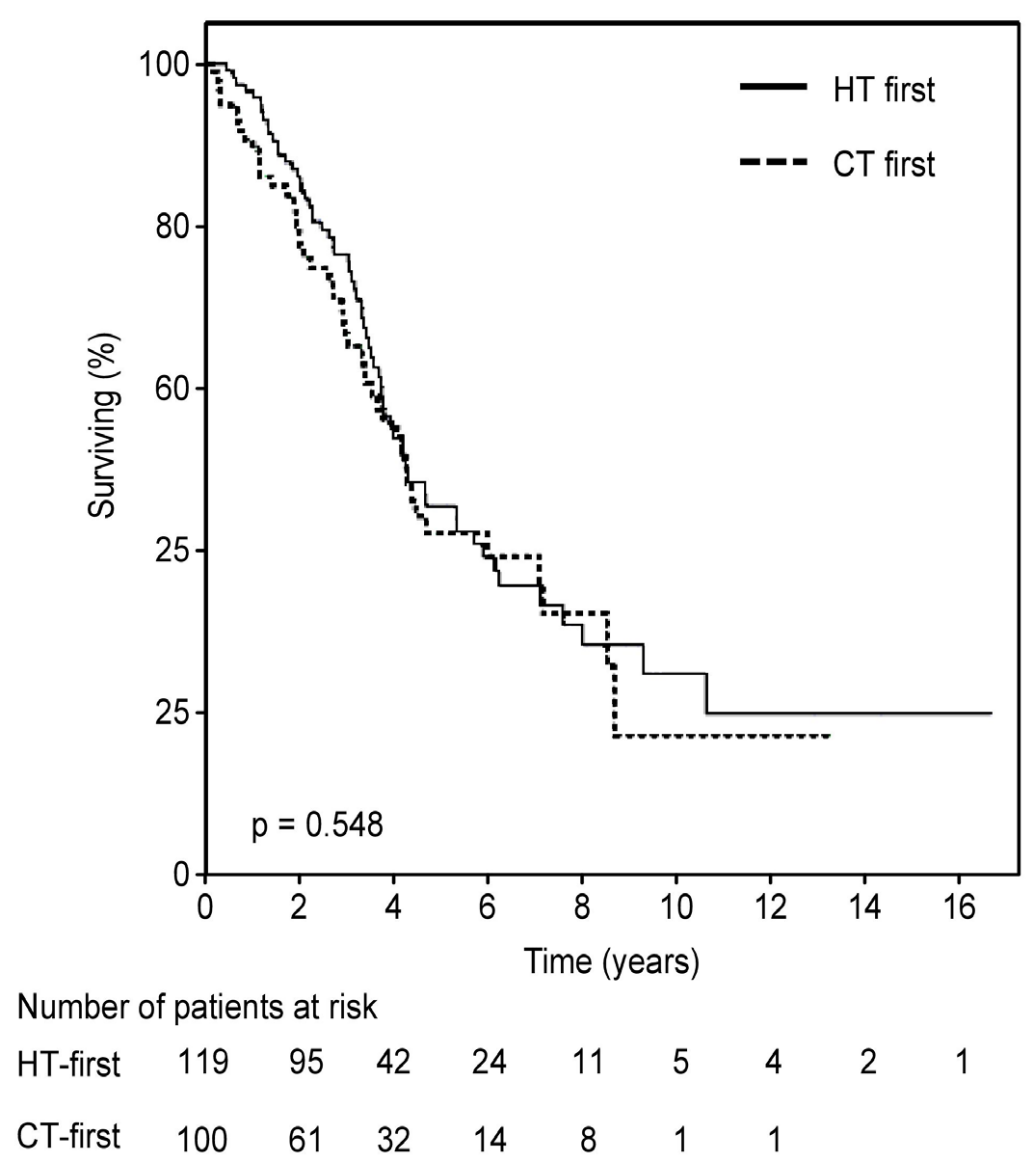
Kaplan-Meier curves or overall survival according to treatment sequence

Overall, 109 deaths were recorded: 62 in the HT-first group and 47 in the CT-first group. Median OS was 50.7 months (95%CI 35.2-66.3) and 51.1 months (95%CI 42.5-59.6) for the HT-first and the CT-first groups, respectively (*p* = 0.548, Figure 2). In the HT-first group, the percentage of surviving patients at 1, 2, 3, 4, and 5 years was 95.8%, 86.1%, 76.5%, 53.9% and 45.5% respectively. The percentages observed in the CT-first group at the same time intervals were 89.5%, 77.4%, 66.7%, 55.7% and 42.1%.

### Time spent in CT

**Figure 3:**
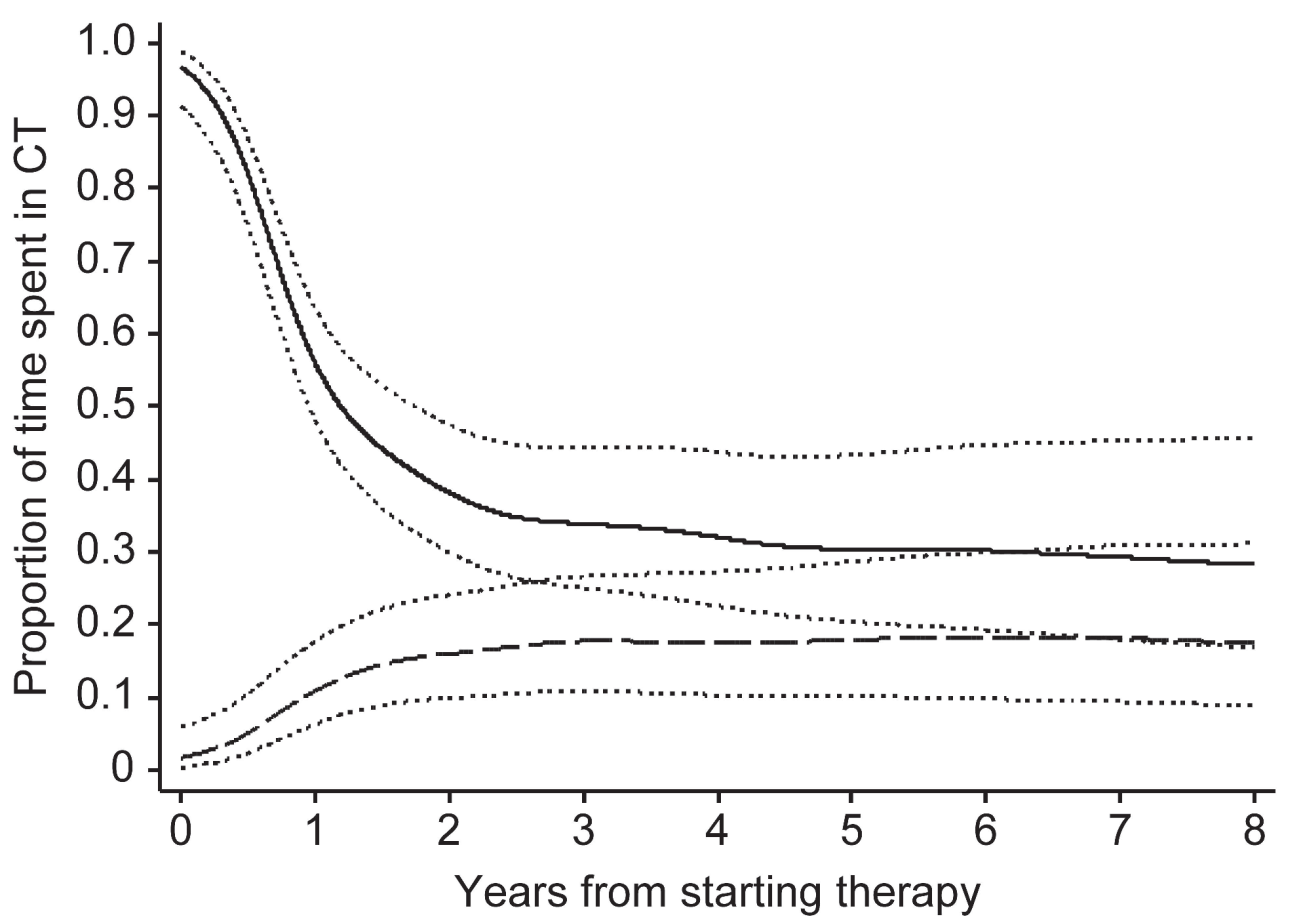
Time spent in CT (chemotherapy) related to OS (overall survival) in HT (hormonal-first group and in CT-first group CT-first group solid line; HT-first group dashed line; 95%CI dot lines.

Figure 3 shows the estimates of the proportion of time/patients spent in CT during the follow-up. Data are truncated at the eighth year from starting therapy because beyond this time the small number of patients prevents to achieve reliable findings. At the beginning of therapy, the proportion of patients in CT in the CT-first group was much higher than in the HT-first group: indeed at the first year the estimates were 0.54 (95% CI 0.46-0.60) and 0.11 (95% CI 0.0-0.18), respectively. Over time, these proportions became closer: 0.34 (95% CI 0.26-0.44) in CT-first group and 0.18 (95% CI 0.11-0.27) in HT-first group within three years and 0.28 (95% CI 0.17-0.45) and 0.18 (95% CI 0.09-0.31) respectively at the end of the investigated period.

### Univariate, multivariate and subgroup analyses

In univariate analyses, the presence of visceral (liver, lung and/or brain) MTS and patients age were significantly associated with OS (*p* = 0.011 and 0.047, respectively). In multivariate analyses, only the presence of visceral MTS was a significant independent predictor of survival (HR = 1.6, 95%CI 1.1-2.4, *p* = 0.022).

**Figure 4:**
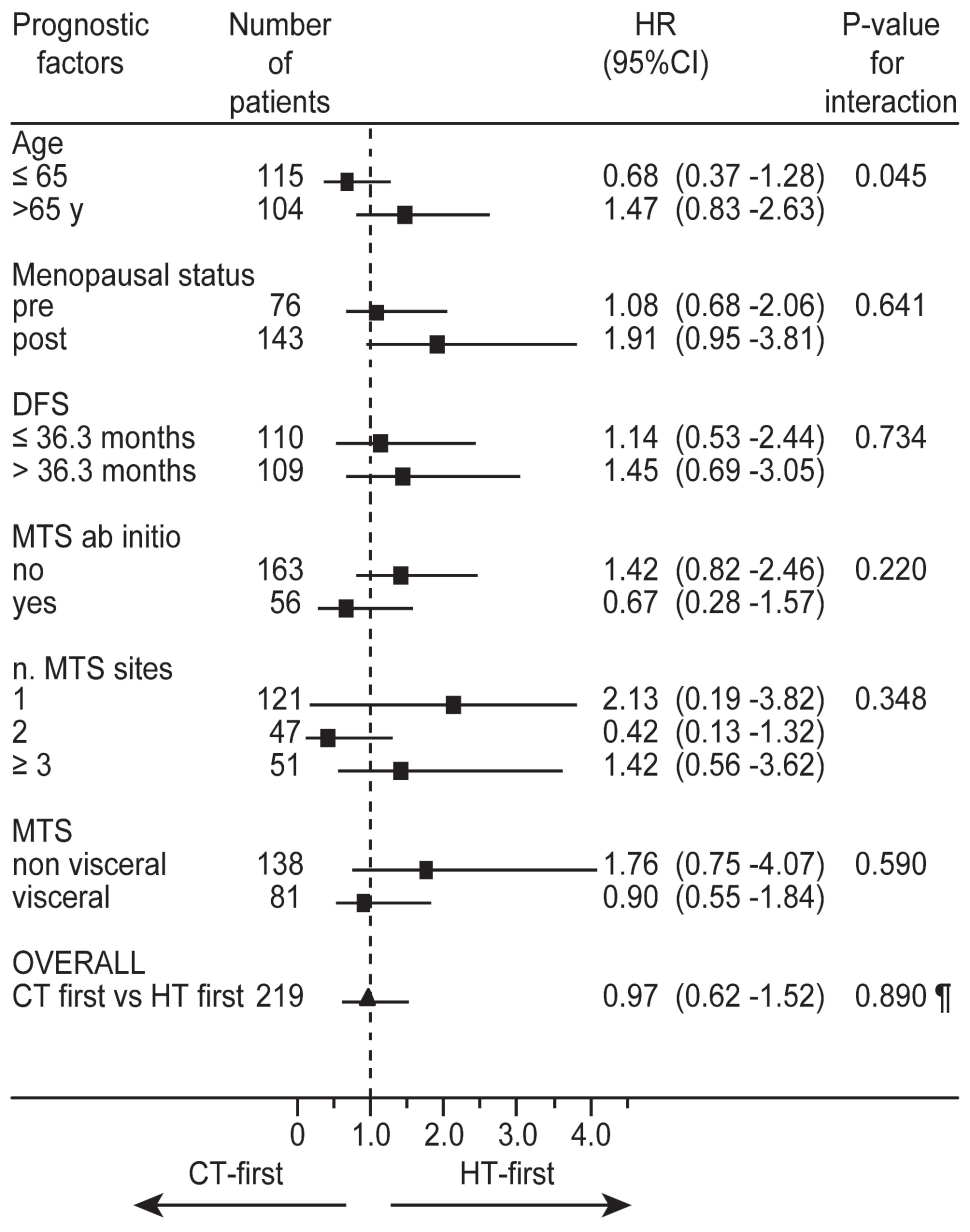
Forest plot of subgroup analyses of overall survival comparing the CT-first sequence versus the HT-first sequence within the strata of each prognostic factor ¶overall comparison of CT-first sequence versus HT-first sequence adjusted for all prognostic factors.

Subgroup analyses of OS comparing the CT-first group versus the HT-first group within the strata of each prognostic factors indicated a possible interaction between treatment sequence and patient age; the significant higher HR of death in patients aged more than 65 years (1.47 versus 0.68, *p* = 0.45) indicates that the HT-first sequence might be more effective in older patients in terms of OS (Figure 4). Conversely, no significant evidence of interaction was observed between the type of treatment sequence and menopausal status, presence of de novo metastatic disease, number of MTS sites, DDFS or presence of visceral or non-visceral MTS. Nevertheless, the HT-first sequence appeared favored in subgroups of patients at lower risk, that is in postmenopausal status, presenting a less aggressive disease (longer DDFS), with single non-visceral MTS.

### Prediction of the use of CT as first-line treatment of MBC

In univariate logistic regressions, the presence of de novo metastatic disease (OR = 2.92, *p* < 0.001), the presence of 2 or ≥3 sites of MTS (OR = 2.05 and 2.91 respectively, *p* = 0.001) and the type of MTS (visceral versus non-visceral, OR = 5.15, *p* < 0.001) were significant predictors of the use of CT as first-line treatment of MBC. On the contrary, older age (>65 years, OR = 0.33, *p* < 0.001), postmenopausal status (OR = 0.40, *p* = 0.001) and long DDFS (OR = 0.48, *p* = 0.0002) were significant predictors of the non-use of CT as first-line therapy (Table 3).

**Table 3:** Prognostic factors associated with a preferential use of CT as first-line treatment of MBC

Prognostic factor	Univariate			Multivariate		
	**OR**	**95% CI**	***p***	**OR**	**95% CI**	***p***
***Age*** ≤65 years >65 years	1 (ref) 0.33	- 0.21-0.53	<0.001	1 (ref) 0.29	- 0.17-0.49	<0.001
***Menopausal status*** premenopause postmenopause	1 (ref) 0.40	- 0.24-0.66	0.004	rfm* -	- -	0.102
***De novo metastatic disease*** no yes	1 (ref) 2.92	- 1.64-5.17	<0.001	1 (ref) 2.89	- 1.54-5.44	0.001
***Number of metastatic sites*** 1 2 ≥3	1 (ref) 2.05 2.91	- 1.17-3.57 1.59-5.34	0.001	rfm - -	- - -	0.400
***Metastasis type*** non-visceral visceral	1 (ref) 5.15	- 3.09-8.57	<0.001	1 (ref) 5.80	- 3.34-10.06	<0.001
***DDFS*** ≤median of 36.3 months >median of 36.3 months	1 (ref) 0.48	- 0.30-0.76	0.002	rfm	- -	0.412

Abbreviations: *rfm, removed from the final model

In multivariate regressions, de novo metastatic disease and MTS type were significant independent predictors of the preferential use of CT as first-line treatment (*p* = 0.001 and *p* < 0.001, respectively) while patient age was significantly associated with the preferred non-use of CT (*p* < 0.001). By contrast menopausal status, a high number of MTS sites and a long DDFS were excluded from the final model (*p* = 0.102, 0.400 and 0.412, respectively).

## DISCUSSION

In our retrospective observational study, we observed that HR+/HER2- MBC patients treated with the sequence, first-line CT followed by HT, as compared with the reverse sequence, despite the poorer prognostic factors, showed a similar median OS of about 51 months. In the first three years, this result was obtained at the price of a longer of OS spent in CT for patients starting with CT as compared to patients treated with a first-line HT but this difference decreased after the third year and overall was 28% in CT-first group and 18% in HT-first group. Type of metastatic disease (visceral versus non-visceral) and younger age are strong predictors of the use of a first-line CT; the presence of visceral metastases is an independent adverse prognostic factor in HR+/HER2- MBC patients.

Our results are consistent with data of the literature showing that younger age and visceral metastases as adverse prognostic factors in HR+/HER2- MBC patients [17–19] and partially support current guidelines that indicate the presence of visceral metastases, although symptomatic, as the main criterion for choosing CT as first-line treatment of these patients [8–10].

Our study supports the results of older randomized trials [11] that showed no differences in terms of efficacy between HT and CT as first-line treatment of HR+ MBC patients. However, the absence of randomization allowed to treat patients at worse prognosis with CT obtaining the same results in terms of survival of the patients with a better prognosis treated with HT. Taking into account the median survival of about 4 years in patients with adverse prognostic factors, the beneficial effect of CT used as first-line treatment in this subgroup of patients cannot be excluded.

Our data are in line with those of a recent study showing that, in real life, a high percentage of patients with HR+ disease receive initial CT in contrast with the recommendation of the current available guidelines [20]. Of note, the overall survival of patients treated with first-line CT in the study by Lobbezoo et al was worse (about 16 months) than the one of our patients ( about 51 months). This difference can be largely explained by the different patients’ characteristics and pattern of cares in two studies (e.g. more bone disease and less multiple metastatic sites in our patients).

One recent observational study comparing CT versus HT as first-line treatment obtained our own results in terms of overall survival (37.5 months and 33.4 months respectively, log-rank test, *P* = 0.62) and progression-free survival (13.3 months and 9.9 months respectively, log-rank test, *P* = 0.92) [21].

Another observational study showed that HR+ MBC patients receive a median of 3 lines of CT with approximately 40% of patients treated with fourth-line CT [22]. Our study confirmed that these patients received a median of 4 (HT-first group) or 3 (CT-first group) lines of treatment for their disease and median durations of HT and CT were the same in the two groups. However, to our knowledge, this is the first study showing that patients treated with CT as first-line treatment spend more time of their survival in CT as compared to patients starting with HT with a potential negative impact on their lives.

The main limitation of our study is its retrospective nature. This factor can lead to several bias including selection of patients and the choice of the treatment. On the other hand, no randomized trial of HT versus CT with treatments including currently used drugs, such as aromatase inhibitors, fulvestrant or taxanes, are available.

Patients included in this study were treated with modern HT and CT but they did not receive the most recently drugs everolimus (used only in two patients) or cyclin-dependent kinase 4/6 inhibitors (palbociclib or ribociclib). The improvements in efficacy obtained in randomized trials from the combination of everolimus and exemestane [23] or palbociclib with letrozole [24] or with fulvestrant [25], or ribociclib plus letrozole [26] could lead in the future to better outcomes with HT and with less use of CT in HR+/HER2- MBC patients.

Moreover, another important limitation is that our study does not answer the question whether HT in maintenance should be given.

In conclusion, our study suggests that HR+/HER2- MBC patients treated with first-line CT followed by HT, despite the poor prognostic factors, had clinical outcomes similar to those of patients treated with the reverse sequence. However, these results are obtained at the price of a longer time of OS spent in CT.
